# Endothelial cell-modified BMSC-GT/PCL nanofiber membrane sheet constructs promote bone tissue regeneration

**DOI:** 10.3389/fbioe.2025.1557279

**Published:** 2025-02-28

**Authors:** Qian Zhou, Mengnan Wen, Yiwu Zhang, Zhinan Wang, Guangdong Zhou, Xiaoqin Liang

**Affiliations:** ^1^ Plastic Surgery Institute, Shandong Second Medical University, Weifang, Shandong, China; ^2^ Department of Plastic and Reconstructive Surgery, Shanghai Key Laboratory of Tissue Engineering, Shanghai Ninth People’s Hospital, Shanghai Jiao Tong University School of Medicine, Shanghai, China; ^3^ Institutes of Health Central Plain, The Third Affiliated Hospital of Xinxiang Medical University, Clinical Medical Center of Tissue Engineering and Regeneration, Xinxiang Medical University, Xinxiang, China

**Keywords:** bone tissue engineering, cell sheet engineering, bone marrow mesenchymal stem cells, endothelial cell modification, GT/PCL nanofiber membrane, bone regeneration

## Abstract

**Introduction:**

Bone defect repair remains a major challenge in modern medicine. Although bone marrow mesenchymal stem cells (BMSCs) possess multilineage differentiation potential, traditional BMSC constructs are often limited in clinical applications due to insufficient osteogenic differentiation efficiency and inadequate vascularization.

**Methods:**

This study developed an innovative bone tissue engineering strategy by combining BMSCs with gelatin/polycaprolactone (GT/PCL) nanofiber membranes to form cell sheets, which were then modified with endothelial cells (ECs) on the surface. The sheets were subsequently rolled into three-dimensional scaffolds to systematically evaluate their osteogenic potential and underlying mechanisms.

**Resuilts:**

Results showed that electrospun GT/PCL nanofiber membranes exhibited uniform fiber structure (diameter 200–500 nm), successfully mimicking the microstructure of natural extracellular matrix. *In vitro* experiments demonstrated that after 14 days of culture, EC modification significantly enhanced the osteogenic differentiation of BMSCs compared to unmodified controls, with approximately 3-fold increase in *ALP* expression (p < 0.05) and 2.5-fold increase in angiogenic factor *VEGF* expression (p < 0.01). Subcutaneous implantation in nude mice revealed superior bone formation capability of EC-modified constructs at both 4 and 8 weeks: micro-CT analysis showed bone density reaching 350 mg/cm^3^, bone surface area approaching 400 mm^2^, and bone volume fraction of approximately 20%, significantly higher than control groups (p < 0.0001). Immunohistochemical evaluation further confirmed more mature trabecular bone structure and richer vascular networks in EC-modified groups.

**Discussion:**

Mechanistic studies revealed that EC modification promoted bone regeneration through three key pathways: optimization of local vascular microenvironment for improved nutrient supply, activation of intercellular synergistic signaling pathways, and reconstruction of physiological bone tissue microenvironment. This study not only validates the application value of this composite strategy in bone tissue engineering but also provides important theoretical basis for developing novel bone regeneration solutions.

## 1 Introduction

Bone tissue engineering and regeneration remain major challenges in contemporary medicine, with treatment strategies continuously evolving to meet clinical demands (Wildemann et al., 2021; Liu et al., 2024). While autologous bone grafts represent the current gold standard for bone regeneration due to their excellent biocompatibility and osteogenic potential, their significant limitations, including donor site morbidity and limited availability, have intensified the search for alternative therapeutic strategies (Wildemann et al., 2021; Wang et al., 2023; Gong et al., 2024). In this context, stem cell-based bone tissue engineering has emerged as a particularly promising approach, offering innovative solutions that address many of these limitations (Liu et al., 2021; Xu et al., 2022; Gao, 2024). Among various stem cell types, bone marrow mesenchymal stem cells (BMSCs) have garnered substantial attention in bone tissue engineering research, primarily due to their remarkable self-renewal capacity, multi-lineage differentiation potential, and particularly their well-documented ability to differentiate into functional osteoblasts (Wang et al., 2024b).

However, BMSC-based approaches face multiple challenges in clinical applications. Traditional BMSC constructs often exhibit limited osteogenic differentiation potential and bone formation efficiency, as well as a lack of appropriate mechanical support and bioactive signaling (Huang et al., 2024). These limitations underscore the need for optimization of BMSC strategies. In response, researchers have developed innovative scaffold systems based on biomaterial nanomaterials.

Among various tissue engineering materials, gelatin/polycaprolactone (GT/PCL) composite nanofiber membranes have emerged as a particularly promising option, offering several unique advantages compared to traditional scaffolds. While pure PCL scaffolds possess excellent mechanical properties and degradation characteristics, they lack cell recognition sites and exhibit hydrophobicity, limiting cell adhesion and proliferation. In contrast, pure gelatin scaffolds demonstrate excellent biocompatibility and cell affinity but suffer from poor mechanical strength and rapid degradation. The GT/PCL composite system successfully overcomes these limitations by combining gelatin’s superior biocompatibility and cell adhesion properties with PCL’s mechanical strength and controllable degradation rate (Fu et al., 2015; Li et al., 2021).

Compared to other widely used tissue engineering materials (such as hydroxyapatite/collagen composites or poly (lactic-co-glycolic acid) (PLGA) scaffolds), GT/PCL nanofiber membranes offer several distinct advantages: 1) their electrospun nanofiber structure (diameter 200–500 nm) more effectively mimics the microstructure of natural extracellular matrix, promoting cell attachment and growth; 2) material composition can be easily adjusted to optimize mechanical properties and degradation rates; 3) the fabrication process allows for easy incorporation of bioactive molecules (Feng et al., 2019; Zheng et al., 2021).

Recent studies have revealed that ECs play a critical role beyond traditional angiogenesis in bone regeneration (Liao et al., 2022; Wang et al., 2024a). By secreting various growth factors and cytokines, ECs can significantly modulate the osteogenic differentiation of BMSCs (Xu et al., 2019; Wang et al., 2024c). This discovery provides new insights into optimizing BMSC-based bone tissue engineering strategies (Qian et al., 2024a). Specifically, the modification of BMSC-material composite sheets with ECs represents an innovative approach, combining the benefits of two distinct cell types while maintaining the structural integrity of the cell sheet (Liang et al., 2019; Qian et al., 2024b).

**SCHEME 1:**
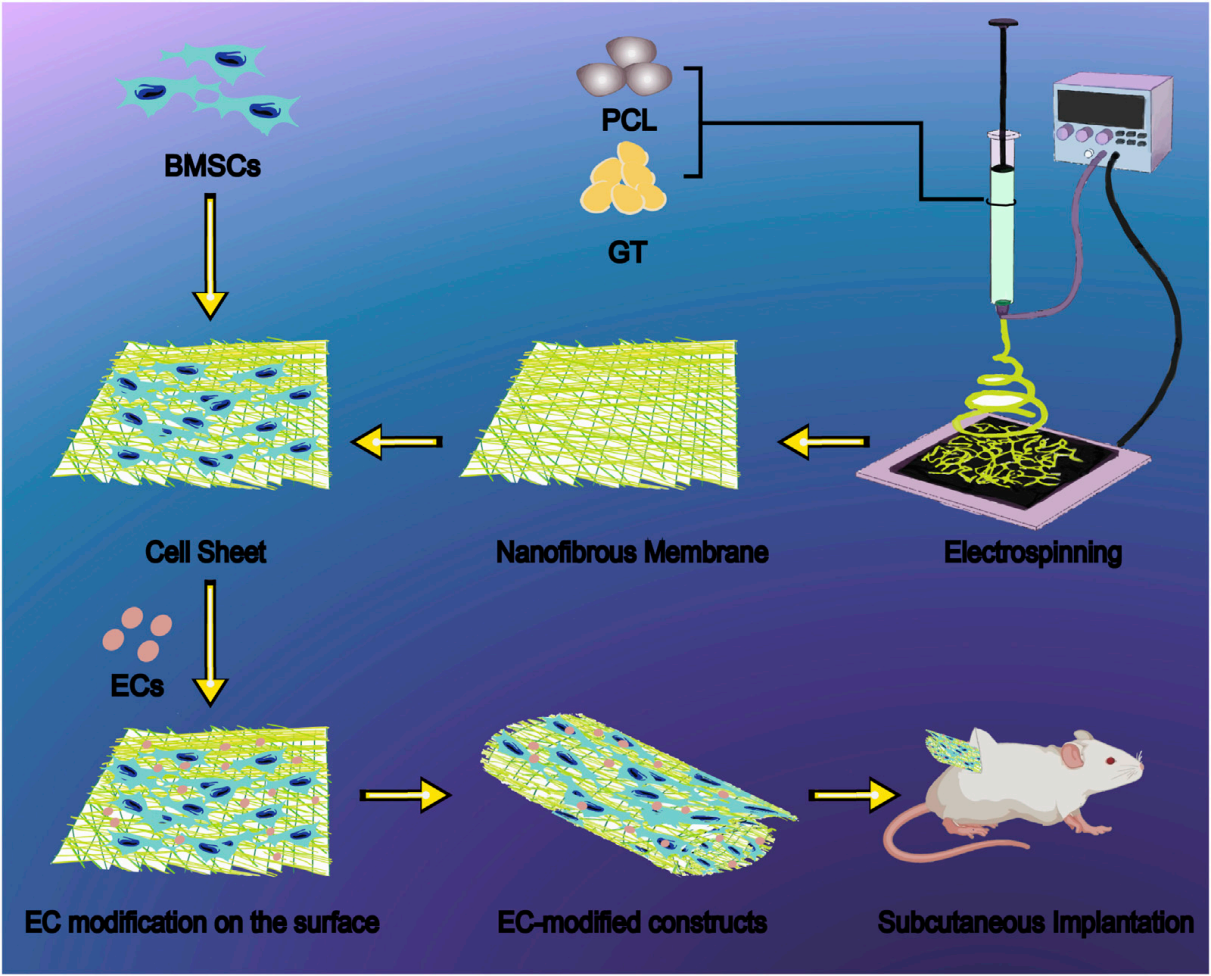
Schematic illustration of endothelial cell-modified BMSC sheet constructs based on GT/PCL nanofiber membranes for bone regeneration. The fabrication process begins with electrospinning of GT/PCL blend solutions to produce uniform nanofibrous membranes with fiber diameters ranging from 200–500 nm, effectively mimicking the natural extracellular matrix architecture. BMSCs are seeded onto the nanofibrous membrane to generate a cell sheet, followed by surface modification with ECs to enhance osteogenic differentiation and vascularization. The resulting composite sheets are rolled into three-dimensional constructs and evaluated through subcutaneous implantation in nude mice, demonstrating superior bone formation capability with increased bone density (350 mg/cm^3^), bone surface area (400 mm^2^), and bone volume fraction (20%) compared to unmodified controls. This innovative bone tissue engineering approach addresses the challenges of insufficient osteogenic differentiation and inadequate vascularization in traditional BMSC constructs. Abbreviations: GT, gelatin; PCL, polycaprolactone; BMSCs, bone marrow mesenchymal stem cells; ECs, endothelial cells.

Based on these advances, we developed an innovative composite strategy by combining BMSCs with GT/PCL nanofiber membranes to form cellular sheets, and then modifying the surface with ECs. We systematically compared the bone regenerative performance of the BMSC-material composite sheets with and without EC modification, focusing on elucidating the mechanisms of cell-material and cell-cell interactions within this composite system (Scheme 1). Our findings deepen the understanding of BMSC-mediated bone regeneration and offer a new paradigm for optimizing bone tissue engineering strategies.

## 2 Materials and methods

### 2.1 Experimental animals and cell lines

In this study, nude mice (6 weeks old) were purchased from Shanghai Slaccas Experimental Animal Ltd., and New Zealand white rabbits (2 kg) were purchased from Shanghai Jiagan Biological Technology Co.

The cell lines present in this study (Primary Umbilical Vein Endothelial Cells; Normal, Human, HUVECs) were obtained from ATCC (Catalog #PCS-100-010™, Manassas, VA, United States).

### 2.2 Material preparation

#### 2.2.1 Preparation of GT/PCL nanofiber membranes

Gelatin (GT, Solarbio Biotech Co., Ltd., China) and polycaprolactone (PCL, Mw80000, Perstorp, Sweden) were mixed in a 3:7 weight ratio and dissolved in hexafluoroisopropanol (HFIP, Sigma, United States) to prepare a 16% (w/v) spinning solution. The solution was stirred at room temperature for 6 h to ensure complete dissolution and mixing. The GT/PCL solution was then loaded into a 10 mL syringe with a 25G blunt needle for electrospinning. The electrospinning parameters were set as follows: feed rate 0.3 mm/min, collection distance 10 cm, and applied voltage 11 kV. The resulting nanofiber membranes were dried under vacuum, followed by chemical crosslinking, cutting, and collection. To ensure sterility, the membranes were sterilized in 75% ethanol for 30 min, washed three times with PBS (5 min each), and finally exposed to ultraviolet light for 30 min on each side.

#### 2.2.2 Isolation and culture of BMSCs

Bone marrow was aspirated from the anterior superior iliac spine of healthy New Zealand rabbits. BMSCs were isolated and cultured in a standard culture medium composed of low-glucose Dulbecco’s Modified Eagle Medium (DMEM, Gibco BRL, Grand Island, NY, United States) supplemented with 10% fetal bovine serum (FBS, Hyclone, Logan, UT, United States), following previously established methods. BMSCs at the second passage (P2) was prepared for the following experiments (Wei et al., 2015; Li et al., 2017).

### 2.3 Construction of cell sheets

#### 2.3.1 Preparation of BMSC sheets

P2 BMSCs were seeded onto pretreated GT/PCL nanofiber membranes at a density of 1.0 × 10^5^ cells/cm^2^, and 50 μg/mL of vitamin C was added to induce cell sheet formation. After 14 days of culture, the BMSC-material composite sheets were gently detached using tweezers.

#### 2.3.2 Endothelial cell modification

Human umbilical vein endothelial cells (HUVECs) were seeded onto the surface of the BMSC-material composite sheets at a density of 1 × 10^4^ cells/cm^2^. Co-culture was performed for 24 h to form the EC-modified BMSC-material composite sheets (EC-modified group, experimental group). Unmodified BMSC-material composite sheets were used as the control group (Control group).

#### 2.3.3 Preparation of composite constructs

The BMSC-material composite sheets from both the Control and EC-modified groups were rolled into cylindrical constructs with a diameter of 3 mm and a height of 8 mm. The steps were as follows: 1) Using sterile tweezers, the composite sheets were rolled along the long axis; 2) Absorbable sutures were used to secure the constructs. The constructed scaffolds were cultured *in vitro* for 14 days before further experiments.

### 2.4 *In vitro* evaluation

#### 2.4.1 Morphological observation

Nanofiber membranes were cut into 1.5 cm diameter discs and placed in 24-well plates. A BMSC suspension of 1.0 × 10^5^ cells/mL was evenly applied to the surface of the membranes. After 24 h of culture, the samples were transferred to new 6-well plates and fixed overnight at 4°C with 0.05% glutaraldehyde. The samples were then dehydrated through graded ethanol and critical-point dried, followed by observation of cell adhesion using scanning electron microscopy (Zeiss Gemini SEM 300, Germany).

BMSCs at a density of 1.0 × 10^5^ cells/mL were seeded onto the membranes and cultured for 1, 4, and 7 days. Cell viability was evaluated using the Calcein-AM/PI live/dead cell staining kit (C542, Dojindo, Shanghai, China).

After 7 days of culture, the BMSC-nanofiber membrane composites were fixed with 4% paraformaldehyde for 30 min and permeabilized with 0.2% Triton X-100 (Aldrich, United States) for 10 min. Rhodamine-conjugated phalloidin (CA1620, Solarbio, Shanghai, China) was used for staining the actin cytoskeleton, and DAPI (C1006, Beyotime, Shanghai, China) was used for nuclear staining. The cell morphology was observed using an inverted fluorescence microscope.

#### 2.4.2 Osteogenic differentiation evaluation

Real-time quantitative PCR (RT-qPCR) for gene expression of osteogenic markers (*ALP*, *VEGF* and *CD31*). Histological staining (HE, Alizarin Red, and COL-1 immunohistochemical staining) to assess mineralized nodule formation and collagen expression.

### 2.5 *In vivo* experiments

#### 2.5.1 Subcutaneous implantation in nude mice

The experimental animals were randomly divided into two groups (n = 4 per group): the Control group (implantation of unmodified BMSC sheets with GT/PCL nanofiber membrane composites) and the EC-modified group (implantation of EC-modified BMSC sheets with GT/PCL nanofiber membrane composites). The constructs were implanted subcutaneously into the dorsal region of nude mice, and samples were collected at 4 and 8 weeks post-implantation for evaluation.

#### 2.5.2 RT-qPCR analysis

Real-time quantitative PCR (RT-qPCR) was performed using *GAPDH* as the internal reference gene to detect the expression levels of osteogenic-related genes (*RUNX2*, *ALP*) and angiogenesis-related genes (*CD31*, *VEGF*). Briefly, total RNA was extracted using TRIzol reagent (Invitrogen, Carlsbad, CA), and the RNA concentration was determined using a Nanodrop 2000 spectrophotometer (Thermo Fisher Scientific, Waltham, MA, United States). Reverse transcription (RT) was performed to obtain cDNA using M-MLV 5 × Reaction Buffer (Promega, Madison, WI, United States), according to previously described methods. RT-qPCR was performed using the SYBR Premix Ex TaqTM II (Takara, Kyoto, Japan), and the results were analyzed using an Applied Biosystems AB instrument (Foster City, CA). All tests were performed in triplicate, normalized relative to the expression of housekeeping gene GADPH, and analyzed using the 2^−ΔΔCT^ method. The primer sequences are provided in the Supplementary Material (Table 1).

**TABLE 1:** The primers sequences of qRT-PCR.

Gene name	Gene identifier	Gene sequence	Product TM [°C]	Product length [bp]
rabbit ALP	M0839bf	5′ CCT TCA CTG CCA TCC TGT AT 3′	86.3	90
	M0839br	5′ GGT AGT TGT TGT GAG CGT AGT C 3′		
rabbit CD31	M1398bf	5′ CCC CGA TCC ATT TCA TAG 3′	82.2	160
	M1398br	5′ ATC CTG ATG CTG ACT TGA CA 3′		
rabbit VEGF	M0206cf	5′ TTA TTT GTA CTG GTT TTT TTG TGT 3′	78.6	87
	M0206cr	5′ GTT CAG GAT AAG CGA GTG AC 3′		
rabbit GAPDH	M0192f	5′ ATG GTG AAG GTC GGA GTG A 3′	83.9	84
	M0192r	5′ AAC ATC CAC TTT GCC AGA GTT A 3′		

#### 2.5.3 Immunohistochemical analysis

The samples were decalcified with 10% EDTA and fixed in 4% paraformaldehyde. After 1 month, the tissues were sectioned longitudinally, dehydrated through graded ethanol, and embedded in paraffin to prepare 3 μm thick sections. Immunohistochemical staining for ALP, OCN, COL-1, and CD31 was performed.

#### 2.5.4 Micro-CT analysis

The samples were fixed in paraformaldehyde and scanned using a VENUS Micro-CT VNC-102 system (PingSheng Medical, Kunshan, China). Three-dimensional reconstructions were performed, and the following parameters were evaluated: bone volume (BV, mm^3^), bone volume fraction (BV/TV), bone surface area (BS, mm^2^), and trabecular mineral density (TMD).

#### 2.5.5 Histological analysis

The samples were sectioned and subjected to HE staining to observe new bone formation and Masson’s trichrome staining to evaluate the bone tissue structure.

### 2.6 Statistical analysis

All quantitative data are expressed as mean ± standard deviation (Mean ± SD). Statistical analyses were performed using SPSS 22.0 software. One-way ANOVA was used for comparisons between groups. A p-value of <0.05 was considered statistically significant.

## 3 Results

### 3.1 Preparation and characterization of GT/PCL nanofiber membranes

**FIGURE 1:**
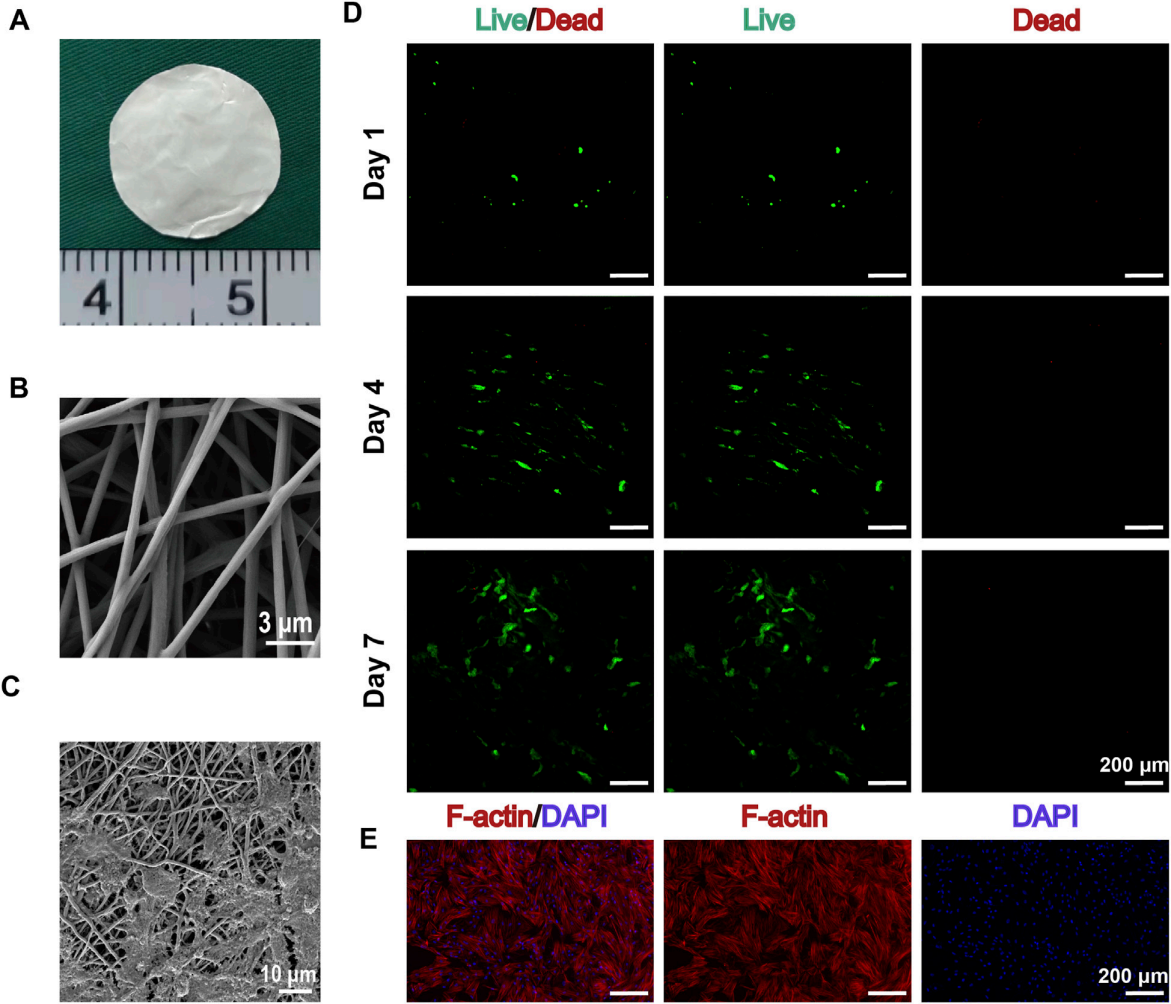
Characterization of GT/PCL Nanofiber Membranes and Their Interaction with BMSCs. **(A)** Macroscopic morphology of GT/PCL nanofiber membranes. **(B)** Scanning electron microscope (SEM) image showing the 3D network structure of electrospun GT/PCL nanofibers, with fiber diameters uniformly distributed in the 200–500 nm range and a smooth surface (scale bar: 3 μm). **(C)** High-magnification SEM image showing the interaction between BMSCs and the nanofiber scaffold, where cells are attached to the fiber network via pseudopodia (scale bar: 10 μm). **(D)** Live/dead cell staining results of BMSCs cultured on GT/PCL nanofiber membranes at days 1, 4, and 7. Green fluorescence indicates live cells, and red fluorescence indicates dead cells. The images show a gradual increase in cell number over time, with excellent cell viability and minimal dead cells (scale bar: 200 μm). **(E)** F-actin/DAPI dual staining showing the cytoskeletal organization of BMSCs. Red fluorescence indicates F-actin fibers, demonstrating well-developed cytoskeletal structure; blue fluorescence represents DAPI-stained cell nuclei. The images show well-spread cells and organized cytoskeletal development, confirming good interaction between the cells and the material (scale bar: 200 μm).

Electrospun GT/PCL nanofiber membranes exhibited excellent characteristics as tissue engineering scaffolds. Scanning electron microscopy (SEM) analysis revealed a highly biomimetic 3D mesh structure, with fiber diameters uniformly distributed in the range of 200–500 nm (Zheng et al., 2021). The surface was smooth and free from breakage or fusion, effectively mimicking the morphological features of natural extracellular matrix (Figures 1A, B). This microstructure provides an ideal microenvironment for cell adhesion and growth.

### 3.2 Cell-material interaction evaluation

#### 3.2.1 Cell adhesion and viability

After 24 h of cell seeding, SEM images showed that BMSCs tightly adhered to the nanofiber membranes through pseudopodia, exhibiting excellent spreading behavior (Figure 1C). Live/dead cell staining results indicated high cell viability and excellent spreading on the scaffold surface. Fluorescence imaging from day 1 to day 7 showed a continuous increase in cell number, predominantly composed of live cells (green fluorescence), with minimal dead cells (red fluorescence) (Figure 1D). Notably, on days 4 and 7, cells showed significant proliferation on the scaffold surface, with a uniform distribution.

#### 3.2.2 Cytoskeletal organization

F-actin/DAPI double staining revealed prominent cytoskeletal development and organization (Figure 1E). Composite fluorescence images demonstrated that cells fully spread on the scaffold surface, displaying organized F-actin fibers (red) and clear nuclear staining (blue), confirming strong interaction between cells and the material. Observation under a 200 μm scale further quantified the extensive coverage of cells on the scaffold surface.

#### 3.2.3 Composite construct formation and histological evaluation

**FIGURE 2:**
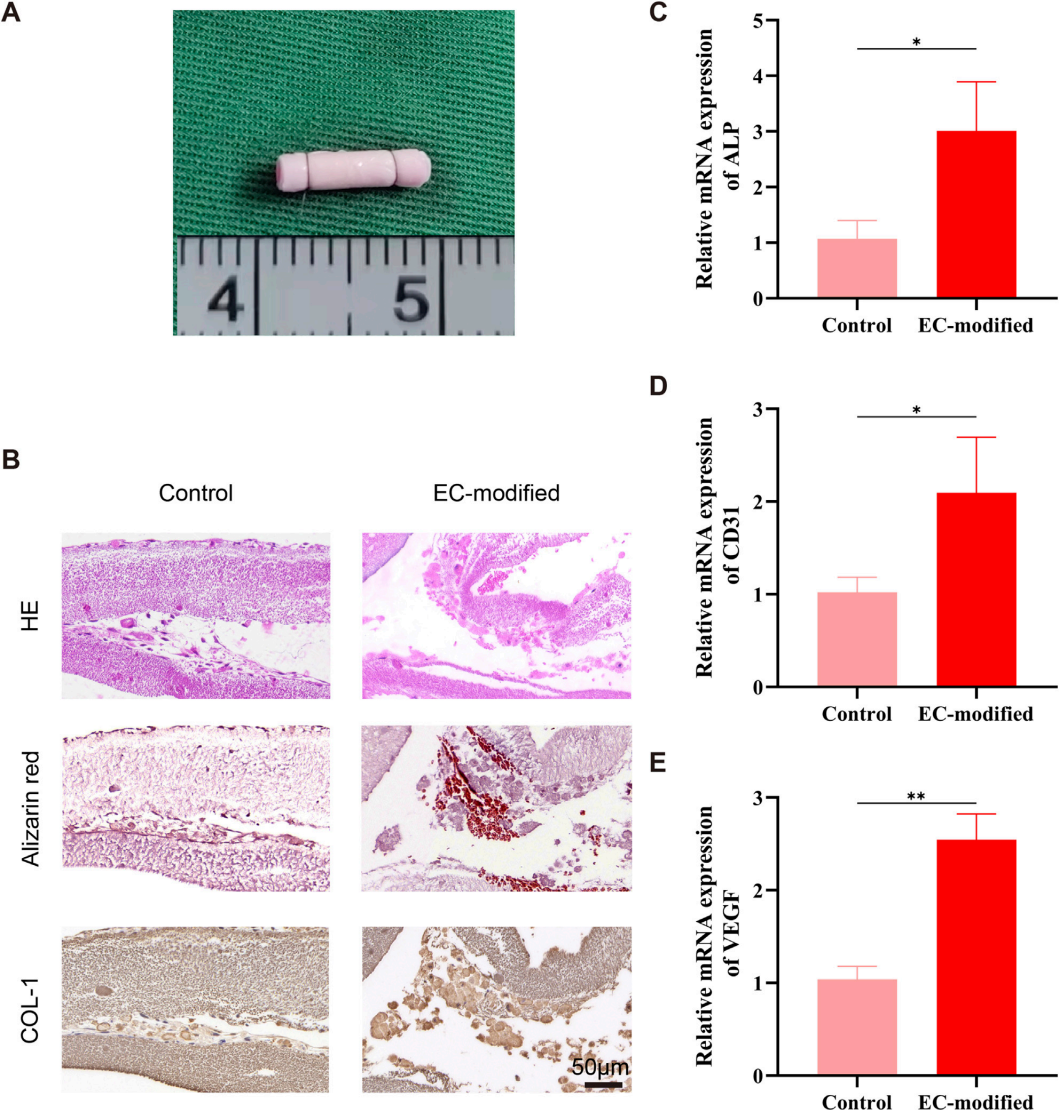
Histological and Molecular Biological Evaluation of Composite Constructs. **(A)** Macroscopic morphology of the composite construct after rolled assembly, presenting a regular cylindrical shape (3 mm in diameter, 8 mm in height). **(B)** Histological evaluation of the composite construct after 14 days of *in vitro* culture. H&E staining (top row) shows cells evenly distributed between the membrane layers, with intact morphology and ordered arrangement; Alizarin red staining (middle row) indicates significant extracellular matrix deposition and mineralization (red positive staining), confirming osteogenic differentiation of BMSCs; Type I collagen immunohistochemical staining (bottom row) shows strong positive expression (brown), further confirming the osteogenic differentiation process (scale bar: 100 μm). **(C–E)** RT-qPCR analysis showing the effect of EC modification on the expression of BMSC differentiation-related genes. Compared to the control group, the EC-modified group exhibited significant upregulation of *ALP* **(C)**, *CD31* **(D)**, and *VEGF* **(E)** mRNA expression levels. Specifically, *ALP* expression increased by approximately 3-fold (p < 0.05), *CD31* expression increased by approximately 2-fold (p < 0.05), and *VEGF* expression increased by approximately 2.5-fold (p < 0.01), indicating that EC modification enhanced both osteogenic differentiation and angiogenesis potential of the composite constructs. Data are presented as mean ± standard deviation, *p < 0.05, **p < 0.01.

After 14 days of vitamin C-induced culture to form BMSC-material composite sheets, constructs were prepared in a rolled-up form, both with and without 24-h endothelial cell (EC) modification, presenting a regular cylindrical shape (3 mm in diameter, 8 mm in height) with a smooth, translucent appearance (Figure 2A). Histological evaluation after 14 days of *in vitro* culture demonstrated excellent cell-material interactions: Hematoxylin and eosin (H&E) staining (Figure 2B, left column) revealed that cells were evenly distributed between the membrane layers, with intact morphology and ordered arrangement in both control and EC-modified groups. Alizarin red staining (Figure 2B, middle column) showed that, compared to the control group, the EC-modified group exhibited not only extracellular matrix deposition but also significantly enhanced mineralized areas (indicated by red positive staining), confirming successful osteogenic differentiation and induction of calcified matrix formation by BMSCs. Immunohistochemical staining for Type I collagen (COL-1) (Figure 2B, right column) demonstrated stronger positive expression in the EC-modified group compared to the control group, further validating the progression of osteogenic differentiation.

### 3.3 *In vitro* biological evaluation

#### 3.3.1 Gene expression analysis

Real-time quantitative PCR results revealed a significant regulatory effect of EC modification on BMSC differentiation potential after 14 days of *in vitro* culture. For osteogenic differentiation-related genes, alkaline phosphatase (*ALP*) expression was significantly elevated in the EC-modified group (p < 0.05), approximately three times higher than in the control group, indicating enhanced osteogenic potential (Figure 2C). This upregulation was consistent with the expression pattern of classic osteogenic markers, suggesting a strengthened trend toward osteoblastic lineage differentiation. For angiogenesis-related genes, the endothelial marker *CD31* showed approximately two-fold increased expression in the EC-modified group (p < 0.05) (Figure 2D), and vascular endothelial growth factor (*VEGF*) expression increased about 2.5-fold (p < 0.01) (Figure 2E). The synergistic upregulation of these two key angiogenic markers further corroborated the enhanced angiogenic potential of the EC-modified group.

### 3.4 *In vivo* bone regeneration evaluation

#### 3.4.1 Macroscopic observation

**FIGURE 3:**
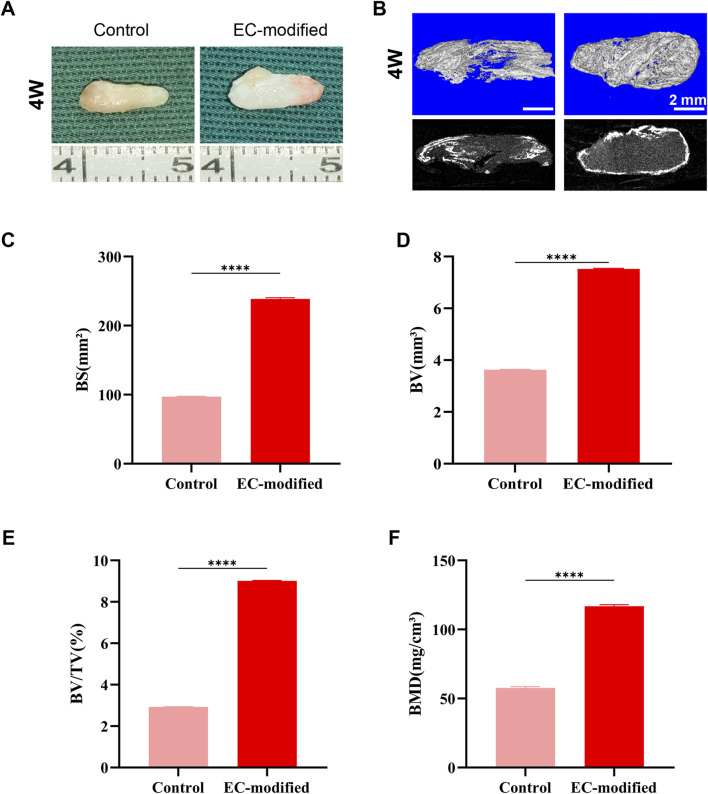
Bone Formation Evaluation of Composite Constructs 4 Weeks Post-Implantation. **(A)** Macroscopic morphology of the composite constructs 4 weeks post-implantation in the subcutaneous tissue of nude mice. Both the Control and EC-modified groups maintained structural integrity and exhibited bone-like tissue characteristics. **(B)** Micro-CT 3D reconstruction images of the implants (top row: color images; bottom row: grayscale images) showing the distribution of mineralized tissue (scale bar: 2 mm). **(C–F)** Quantitative analysis based on Micro-CT: **(C)** Bone surface area (BS) in the EC-modified group was significantly higher than in the Control group, approximately 240 mm^2^; **(D)** Bone volume (BV) in the EC-modified group was approximately 7.5 mm^3^; **(E)** Bone volume fraction (BV/TV) in the EC-modified group was approximately 9%; **(F)** Bone mineral density (BMD) in the EC-modified group reached approximately 120 mg/cm^3^. Significant differences were observed in all parameters between the groups (**p < 0.0001). Data are presented as mean ± standard deviation.

**FIGURE 4:**
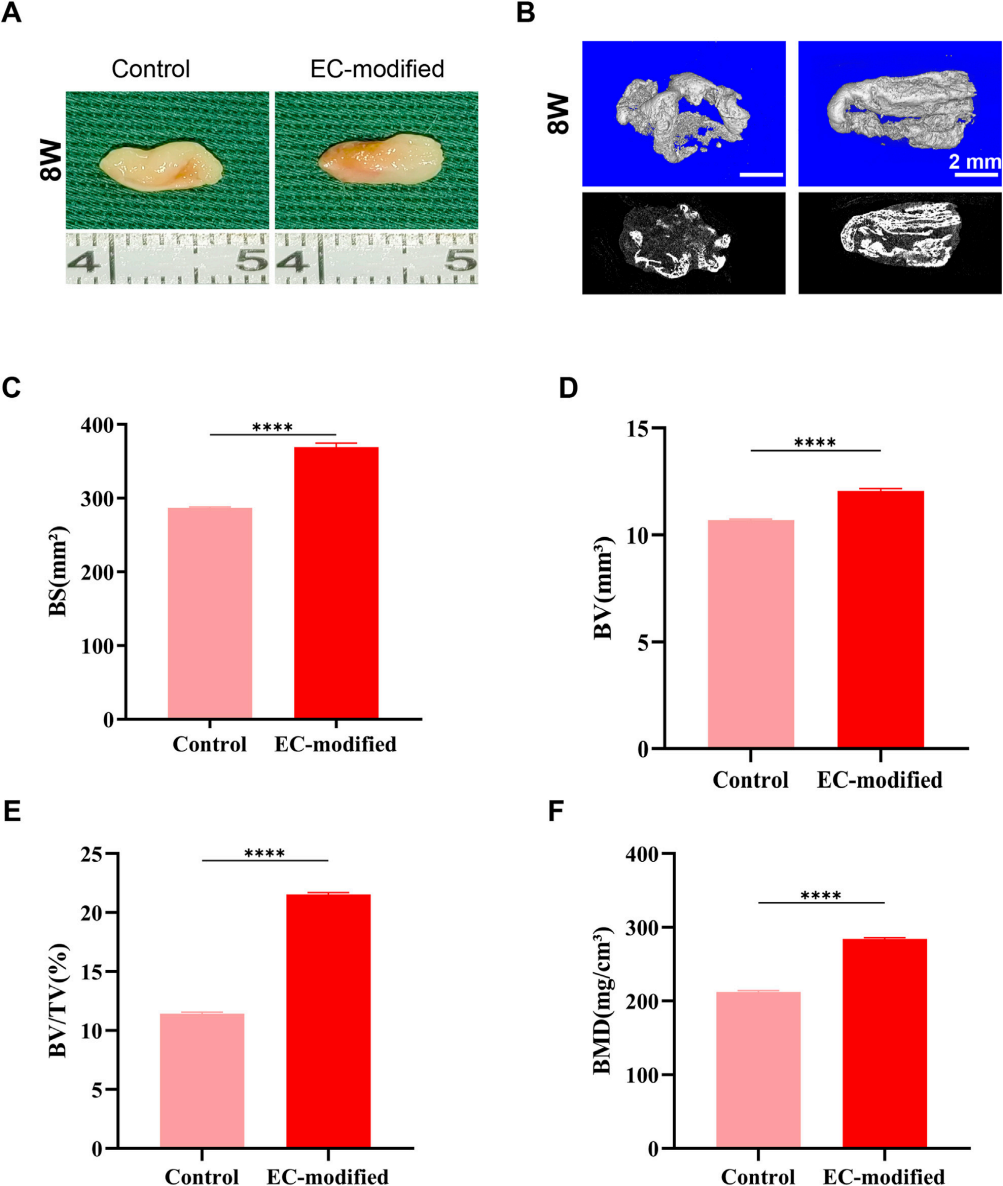
Bone Formation Evaluation of Composite Constructs 8 Weeks Post-Implantation. **(A)** Macroscopic morphology of the composite constructs 8 weeks post-implantation in the subcutaneous tissue of nude mice. Both groups maintained good structural integrity and continued to exhibit obvious bone-like tissue characteristics. **(B)** Micro-CT 3D reconstruction images of the implants (top row: color images; bottom row: grayscale images) showing greater mineralized tissue formation (scale bar: 2 mm). **(C–F)** Quantitative analysis based on Micro-CT: **(C)** Bone surface area (BS) in the EC-modified group approached 400 mm^2^; **(D)** Bone volume (BV) in the EC-modified group significantly increased to approximately 12 mm^3^; **(E)** Bone volume fraction (BV/TV) in the EC-modified group increased to approximately 20%; **(F)** Bone mineral density (BMD) in the EC-modified group reached approximately 280 mg/cm^3^. All bone formation parameters were significantly higher in the EC-modified group compared to the Control group (**p < 0.0001). Data are presented as mean ± standard deviation.

Macroscopic observations of the constructs implanted subcutaneously in nude mice at 4 and 8 weeks (Figures 3A, 4A) showed that all samples maintained good morphological integrity. Both the control and EC-modified groups exhibited features resembling natural bone tissue, indicating that the constructs supported bone tissue regeneration *in vivo*.

#### 3.4.2 Micro-CT analysis

Micro-CT scanning and three-dimensional reconstruction analysis (Figures 3B, 4B) were used to quantitatively assess new bone formation. The 3D reconstructed images showed that the EC-modified group formed more mineralized tissue compared to the control group. Quantitative analysis revealed the following results: For bone mineral density (BMD), the EC-modified group had significantly higher values at both the 4-week and 8-week time points, reaching approximately 120 mg/cm^3^ at 4 weeks (Figure 3F) and increasing to around 280 mg/cm^3^ at 8 weeks (Figure 4F). Regarding bone surface area (BS), the EC-modified group exhibited larger values at both time points, with approximately 240 mm^2^ at 4 weeks (Figure 3C) and nearly 400 mm^2^ at 8 weeks (Figure 4C). For bone volume (BV), the EC-modified group had a value of approximately 7.5 mm^3^ at 4 weeks (Figure 3D), which significantly increased to around 12 mm^3^ at 8 weeks (Figure 4D). The bone volume fraction (BV/TV) of the EC-modified group showed a significantly higher relative bone volume percentage compared to the control group, reaching approximately 9% at 4 weeks (Figure 3E) and increasing to about 20% at 8 weeks (Figure 4E). All parameters showed statistically significant differences between groups at both 4-week and 8-week time points (p < 0.0001).

#### 3.4.3 Histological evaluation

**FIGURE 5:**
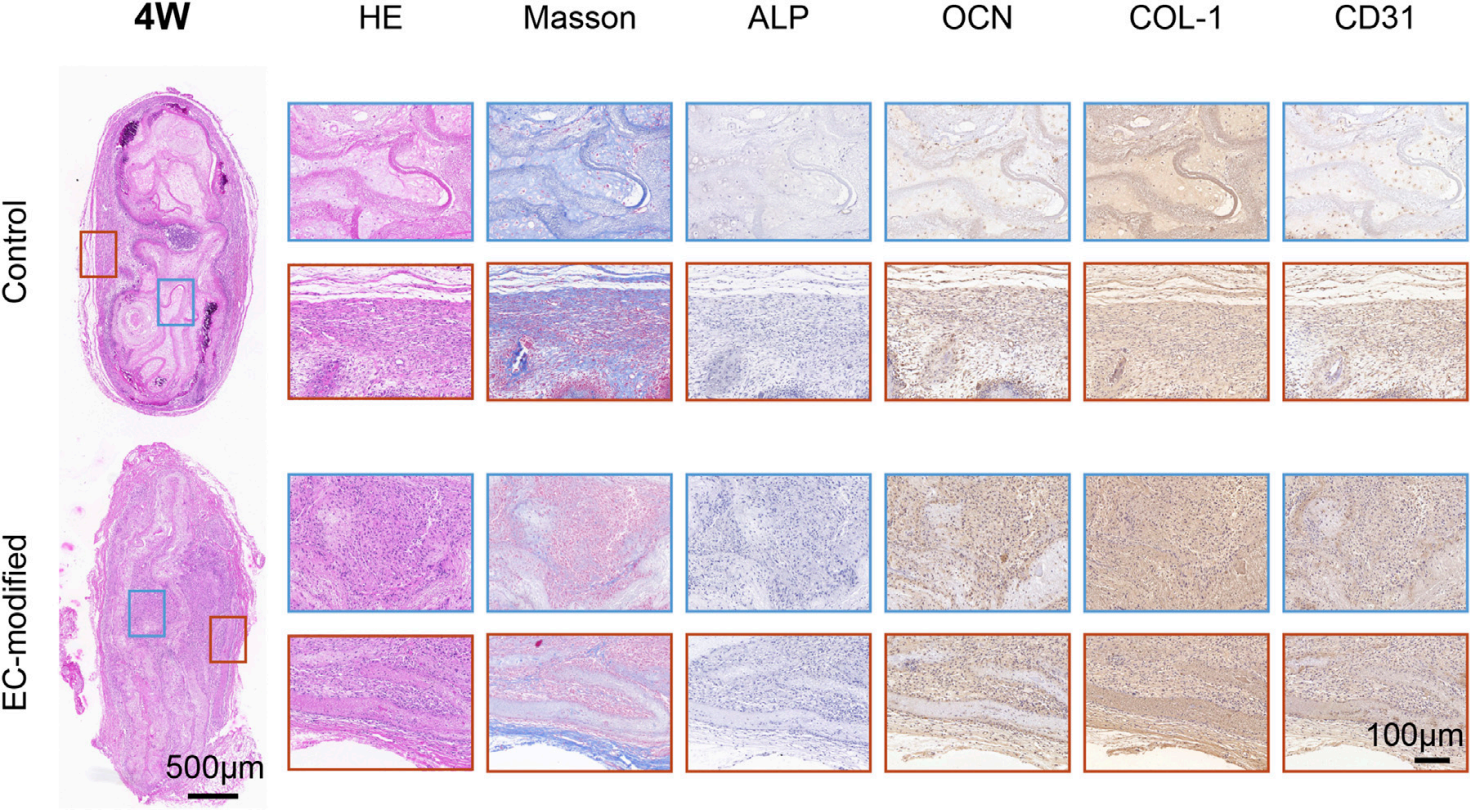
Histological and Immunohistochemical Analysis of Implanted Materials in the Control and EC-Modified Groups (4 Weeks). Overall tissue sections 4 weeks post-implantation (scale bar = 500 μm), with red and blue boxes indicating the regions for high-magnification images. Tissue sections were evaluated by H&E staining, Masson trichrome staining, and immunohistochemical staining for ALP, OCN, COL-1, and CD31 (scale bar = 100 μm). H&E staining revealed tissue morphology, Masson staining highlighted collagen fiber deposition in blue, and immunohistochemical staining showed the expression distribution of various markers (ALP, OCN, COL-1, and CD31). The EC-modified group exhibited stronger staining intensity and more widespread positive expression regions, indicating enhanced bone tissue regeneration and angiogenesis potential.

**FIGURE 6:**
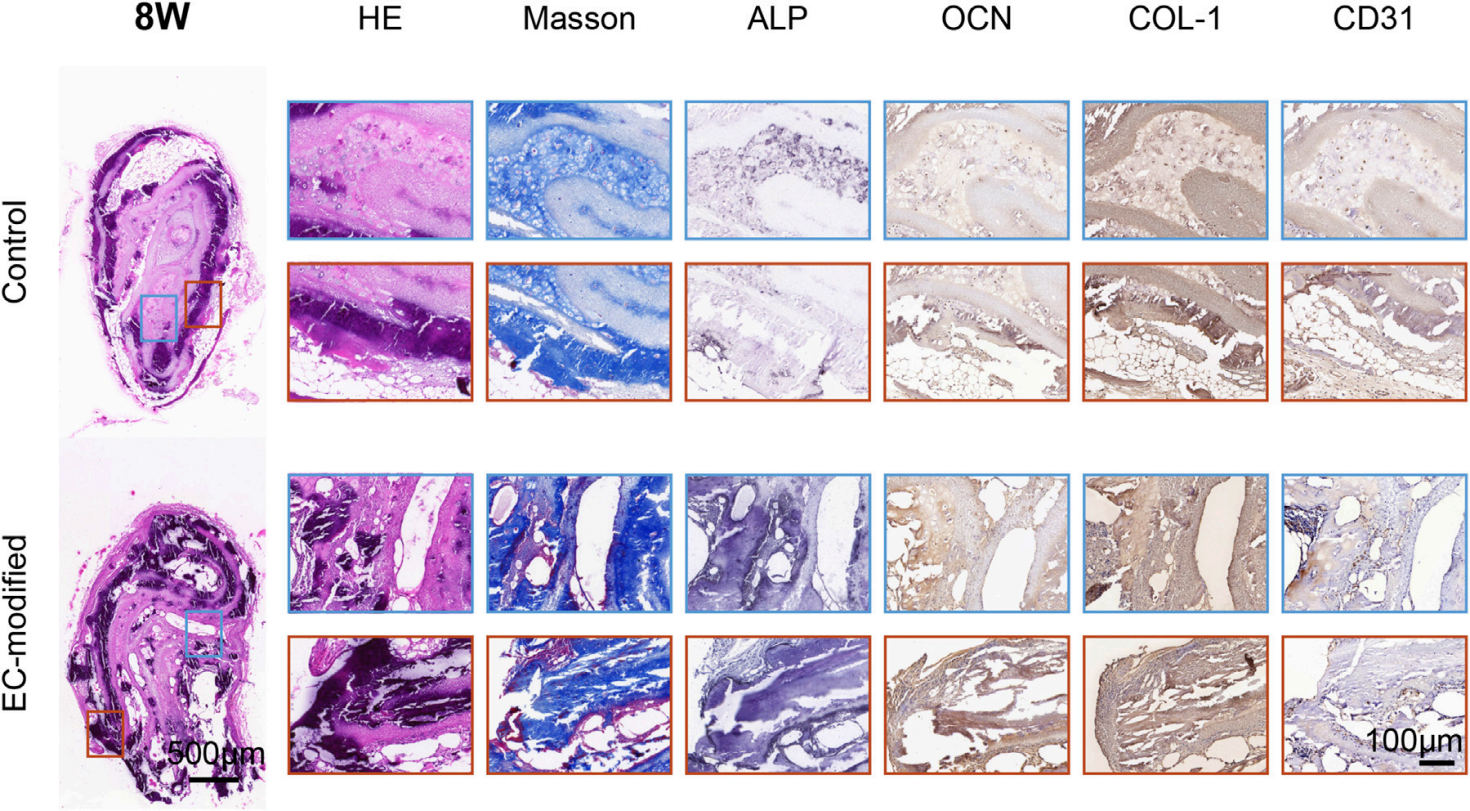
Histological and Immunohistochemical Analysis of Implanted Materials in the Control and EC-Modified Groups (8 Weeks). Overall tissue sections 8 weeks post-implantation (scale bar = 500 μm), with red and blue boxes indicating regions for high-magnification images. The tissue sections were processed using the same staining methods (scale bar = 100 μm). Compared to 4 weeks, the EC-modified group at 8 weeks exhibited more mature bone tissue structures, including denser trabecular bone and more abundant collagen deposition (Masson staining). Immunohistochemical results showed sustained upregulation of bone formation markers (ALP, OCN, COL-1) and angiogenesis markers (CD31) in the EC-modified group, further confirming its superior bone tissue regeneration and vascularization potential.

H&E staining demonstrated superior bone formation in the EC-modified group at both 4 and 8 weeks post-implantation (Figures 5, 6). At 4 weeks, the EC-modified group showed more extensive new bone formation and better-defined bone matrix structure. This advantage became even more pronounced at 8 weeks, where the EC-modified group exhibited more mature bone tissue features, including well-organized trabecular bone structure and higher bone density. Masson’s trichrome staining further confirmed the superior maturity of bone tissue in the EC-modified group (Figures 5, 6). Stronger blue staining, indicating more abundant collagen deposition and more organized bone matrix formation, was observed. This difference was particularly evident at 8 weeks, where the EC-modified group displayed a denser and more mature bone tissue structure, with a more compact collagen fiber network.

#### 3.4.4 Immunohistochemical analysis

Immunohistochemical evaluations showed significantly enhanced expression of osteogenic markers in the EC-modified group (Figures 5, 6). ALP expression was higher in the EC-modified group at both time points, with a gradual increase from 4 to 8 weeks, indicating sustained osteogenic activity. Osteocalcin (OCN) immunostaining revealed stronger positive expression in the EC-modified group, especially at 8 weeks, indicating greater matrix mineralization and maturation compared to the control group. Type I collagen (COL-1) expression was significantly elevated in the EC-modified group, with stronger and more widespread staining at both time points, confirming enhanced matrix protein synthesis and tissue remodeling.

In terms of vascularization, CD31 immunostaining demonstrated a clear angiogenic advantage in the EC-modified group. At 4 weeks, more CD31-positive blood vessels were observed in the EC-modified group compared to the control group. By 8 weeks, this difference became more pronounced, with the EC-modified group exhibiting a more extensive and mature vascular network. Enhanced vascularization in the EC-modified group suggested better nutrient and oxygen supply to the regenerating bone tissue, potentially contributing to improved bone formation (Figures 5, 6; Supplementary Figure S1).

#### 3.4.5 Gene expression analysis

**FIGURE 7:**
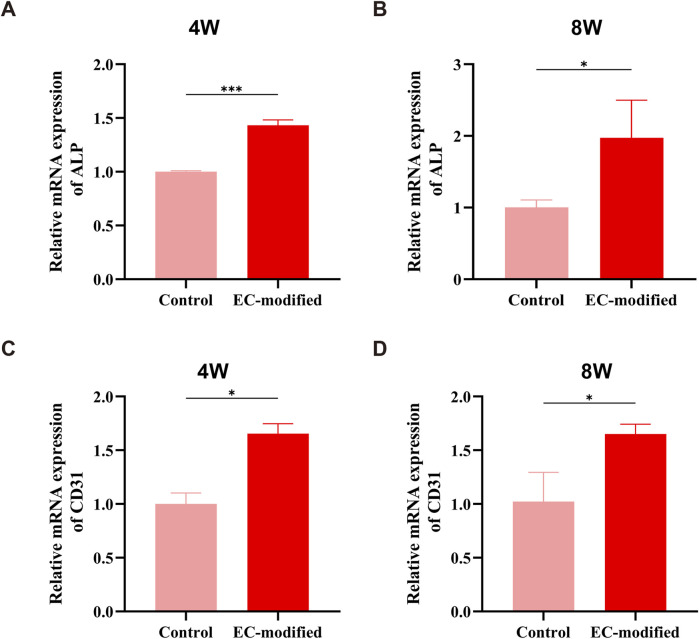
Osteogenesis and Angiogenesis-Related Gene Expression in the EC-Modified and Control Groups at Different Time Points. **(A, B)** RT-qPCR analysis of the relative expression levels of the *ALP* gene at 4 and 8 weeks post-implantation. At 4 weeks, the EC-modified group showed a higher expression trend than the Control group (***P < 0.001); at 8 weeks, the *ALP* expression in the EC-modified group was significantly higher (*P < 0.05), indicating a continuous enhancement of osteogenic differentiation. **(C, D)** *CD31* gene expression analysis revealed a significant angiogenesis advantage in the EC-modified group at both time points. At 4 and 8 weeks, the EC-modified group showed significantly higher *CD31* expression than the Control group (*P < 0.05), confirming the ongoing vascularization process. All data are presented as mean ± standard deviation (n = 3), normalized to the Control group expression level.

Real-time quantitative PCR (RT-qPCR) analysis revealed a significant impact of endothelial cell modification on the gene expression profile of BMSC membrane sheets. The expression of the osteogenic differentiation marker *ALP* exhibited a clear time-dependent increase: At 4 weeks post-implantation, the EC-modified group showed a significant 1.4-fold increase compared to the control group (Figure 7A, p < 0.001). This advantage was further enhanced at 8 weeks, with *ALP* expression levels in the EC-modified group reaching approximately twice that of the control group (Figure 7B, p < 0.05). The sustained increase in *ALP* expression not only indicated a significant enhancement in osteogenic differentiation capacity but also reflected the stability of the osteogenic differentiation process.

The expression pattern of the angiogenic marker *CD31* also demonstrated significant features: At 4 weeks post-implantation, the EC-modified group exhibited approximately 1.7 times higher *CD31* expression compared to the control group (Figure 7C, p < 0.05). This difference was maintained at 8 weeks, with the EC-modified group retaining approximately 1.6 times higher expression (Figure 7D, p < 0.05). The sustained high levels of *CD31* expression not only confirmed the advantage of the EC-modified group in angiogenesis but also indicated the stability and continuity of the vascularization process.

These gene expression data were well corroborated by histological observations: The upregulation of *ALP* expression corresponded to enhanced osteogenic differentiation observed in histological sections, while the sustained high expression of *CD31* supported the formation of a vascular network as shown by immunohistochemical staining. The temporal changes in gene expression were consistent with the dynamic progression of tissue reconstruction.

### 3.5 Comprehensive analysis

Multidimensional evaluation results consistently indicated that endothelial cell (EC) modification significantly enhanced the osteogenic and angiogenic potential of BMSCs. This enhancement exhibited a time-dependent effect, with the most pronounced improvements observed at 8 weeks. The increased expression of markers observed via immunohistochemistry, gene upregulation confirmed by RT-qPCR, and structural improvements seen in histological observations all corroborate each other, collectively supporting the superiority of EC-modified BMSC-material composite constructs in bone defect repair. Notably, the formation and maturation of the vascular network provided a favorable microenvironment for tissue regeneration, which may represent a key mechanism driving enhanced bone tissue regeneration (Liu et al., 2020; Zhao et al., 2025; Zhang et al., 2023).

## 4 Discussion

This study developed an innovative bone tissue engineering strategy: BMSCs were cultured with GT/PCL nanofiber membranes to form BMSC-material composite sheets, followed by EC surface modification, and finally, a 3D scaffold was constructed using a rolling-folding technique. Systematic *in vitro* and *in vivo* experimental results revealed significant advantages of this composite strategy in enhancing bone regeneration, while also elucidating its underlying mechanisms.

In terms of material design, GT/PCL nanofiber membranes exhibited ideal scaffold properties. The electrospun nanofiber structure (200–500 nm) successfully mimicked the morphological characteristics of the natural extracellular matrix, providing an optimized 3D microenvironment for BMSC growth and functional expression (Kim et al., 2017; Zhang et al., 2024). Scanning electron microscopy (SEM) observations showed uniform fiber distribution, smooth and intact surfaces, with no breakage or fusion, and this structural feature closely resembled the microarchitecture of natural bone tissue, effectively promoting BMSC adhesion and proliferation. Live/dead cell staining and F-actin/DAPI double staining further confirmed the excellent biocompatibility of the material, with high cell survival, good spreading, and organized cytoskeletal structures.

Next, *in vitro* results revealed the significant regulatory effect of EC modification on the BMSC-material composite sheet. Real-time PCR analysis after 14 days of culture showed that, compared to the control group, the expression of osteogenic marker *ALP* in the EC-modified group was approximately three times higher (p < 0.05), while angiogenesis-related markers *CD31* and *VEGF* increased by about two times (p < 0.05) and 2.5 times (p < 0.01), respectively. This synergistic upregulation suggests that endothelial cells not only enhance the osteogenic differentiation potential of BMSCs but also promote angiogenesis, forming a positive feedback regulatory network conducive to bone regeneration (Zhu et al., 2019). Histological analysis further supported this conclusion, with HE staining and Alizarin red staining showing better cell morphology and more calcium nodule formation, while COL-1 immunohistochemical staining confirmed the significant enhancement of collagen type I expression.

The most striking results were observed in the *in vivo* experiments. Micro-CT analysis showed that at both 4 and 8 weeks post-implantation, the EC-modified group exhibited significantly superior bone formation compared to the control group. Specifically, this was evidenced by higher bone mineral density (BMD), reaching approximately 350 mg/cm^3^ at 8 weeks, larger bone surface area (BS), approaching 400 mm^2^ at 8 weeks, and a higher bone volume fraction (BV/TV), reaching around 20% at 8 weeks. The significant improvement in these quantitative parameters (p < 0.0001) strongly confirmed the promoting effect of EC modification on bone tissue regeneration. Histological evaluation and immunohistochemical analysis further validated this advantage, showing more mature trabecular structures, higher bone density, and a more extensive vascular network. Notably, the vascular density in the EC-modified group was significantly higher than in the control group, with this angiogenic advantage becoming apparent at 4 weeks and becoming more pronounced at 8 weeks. This finding was in excellent agreement with the gene expression analysis results: *CD31* showed a significant upregulation at 4 weeks (p < 0.01), and the difference expanded further at 8 weeks (p < 0.001); meanwhile, *ALP* expression continued to rise, reaching a significant difference at 8 weeks (p < 0.01). This synergistic enhancement of angiogenesis and osteogenic differentiation was evident not only at the molecular level but also in histomorphological features, such as the denser collagen fiber network shown by Masson staining and stronger expression of osteocalcin (OCN) and Type I collagen (COL-1) confirmed by immunohistochemistry. These results collectively demonstrate that endothelial cells not only successfully participated in and enhanced the formation of new blood vessels but also promoted bone tissue maturation and reconstruction by improving the microcirculation environment (Zhuang et al., 2023).

Based on these experimental results, we propose that the enhanced bone regeneration observed in EC-modified constructs can be attributed to several key mechanisms: First, adaptation to and modification of the subcutaneous microenvironment: While the subcutaneous space is not a typical osteogenic site, our results demonstrate successful bone formation in this ectopic location. We hypothesize that the increased vasculature created by endothelial cells not only improved oxygen and nutrient supply but also established a specialized microenvironment that supported osteogenic differentiation (Li et al., 2023; Lee et al., 2024). This environment likely mimicked aspects of the bone marrow niche through the secretion of specific growth factors and matrix proteins. The sustained high expression of CD31 (p < 0.001 at 8 weeks) observed in RT-qPCR analysis supports the establishment of this stable, pro-osteogenic niche. Second, complex bidirectional interactions between endothelial cells and BMSCs: Our findings reveal sophisticated crosstalk between HUVECs and BMSCs. The significant upregulation of VEGF (increased by 2.5 times compared to the control group) indicates active paracrine signaling from endothelial cells to BMSCs. This interaction likely triggered multiple pathways: 1) endothelial cells secreted osteogenic factors such as BMP-2 and endothelin-1, which promoted BMSC differentiation (Chen et al., 2018; Zhang et al., 2020); 2) BMSCs, in turn, produced angiogenic factors that supported endothelial cell survival and function (Velayutham et al., 2024); and 3) direct cell-cell contact through mechanisms like Notch signaling may have further enhanced osteogenic differentiation. This reciprocal relationship created a positive feedback loop that enhanced bone regeneration (Dai et al., 2021). Finally, establishment of a bone-mimetic cellular ecosystem: Despite the subcutaneous location, the combination of endothelial cells and BMSCs successfully created a microenvironment that recapitulated key aspects of natural bone tissue. The endothelial cells not only provided vascular support but also contributed to the establishment of a bone-like stem cell niche through the secretion of specific extracellular matrix proteins and growth factors. This environmental conditioning likely enabled BMSCs to undergo osteogenic differentiation even in this ectopic location. The enhanced expression of osteogenic markers ALP, OCN, and COL-1, as demonstrated in immunohistochemical analysis, confirms the successful establishment of this bone-mimetic environment (You et al., 2024).

In summary, our findings not only confirm the effectiveness of endothelial cell modification in bone tissue engineering but also provide novel insights into the mechanisms of BMSC-EC interactions in a three-dimensional construct. The successful osteogenic differentiation in a subcutaneous environment demonstrates the robustness of our strategy and its potential for broader clinical applications. However, there are still areas that require further investigation in future studies, such as optimizing the density of endothelial cell modification, assessing the long-term stability of the constructs, and exploring the molecular mechanisms of intercellular communication.

## 5 Conclusion

In this study, we successfully developed an innovative bone tissue engineering strategy by culturing BMSCs with GT/PCL nanofiber membranes to form BMSC-material composite sheets, followed by endothelial cell surface modification, and constructing a 3D scaffold using the rolling-folding technique. Through systematic *in vitro* and *in vivo* experiments, we demonstrated that endothelial cell modification significantly enhanced the osteogenic potential and angiogenesis of BMSC-material composite constructs. Compared to the control group, the EC-modified group exhibited superior bone formation and vascular network development, characterized by higher bone volume fraction, enhanced trabecular parameters, and doubled vascular density. These effects can be attributed to multiple synergistic mechanisms, including optimized vascular microenvironment, activated intercellular signaling pathways, and improved physiological cell composition.

## Data Availability

The raw data supporting the conclusions of this article will be made available by the authors, without undue reservation.
