# Supplementary material for: Endothelial cell-modified BMSC-GT/PCL nanofiber membrane sheet constructs promote bone tissue regeneration
Source: Front Bioeng Biotechnol. 2025 Feb 28;13:1557279. doi: 10.3389/fbioe.2025.1557279 (PMC11906688; doi:10.3389/fbioe.2025.1557279)
Supplement: Supplementary file 1 [file DataSheet1.docx]

Endothelial Cell-Modified BMSC-GT/PCL Nanofiber Membrane Sheet Constructs Promote Bone Tissue Regeneration

Qian Zhou ^1,2†^, Mengnan Wen ^3†^, Yiwu Zhang ^1^, Zhinan Wang ^1^, Guangdong Zhou ^2^*, and Xiaoqin Liang ^1^*

^1^ Plastic Surgery Institute, Shandong Second Medical University, Weifang, Shandong 261053, PR China

^2^ Department of Plastic and Reconstructive Surgery, Shanghai Key Laboratory of Tissue Engineering, Shanghai Ninth People's Hospital, Shanghai Jiao Tong University School of Medicine, Shanghai 200011, PR China.

^3^ Institutes of Health Central Plain, The Third Affiliated Hospital of Xinxiang Medical University, Clinical Medical Center of Tissue Engineering and Regeneration, Xinxiang Medical University, Xinxiang 453003, PR China

† These authors contributed equally to this work.

*** Correspondence:**

E-mail: [guangdongzhou@126.com](mailto:guangdongzhou@126.com) (Guangdong Zhou);

[liangxq2002@163.com](mailto:liangxq2002@163.com) (Xiaoqin Liang)

Keywords: bone tissue engineering, cell sheet engineering, bone marrow mesenchymal stem cells, endothelial cell modification, GT/PCL nanofiber membrane, bone regeneration.

Table 1. The primers sequences of qRT-PCR.

| Gene Name | Gene Identifier | Gene Sequence | Product TM  [°C] | Product Length  [bp] |
| --- | --- | --- | --- | --- |
| rabbit ALP  rabbit CD31  rabbit VEGF  rabbit GAPDH | M0839bf  M0839br  M1398bf  M1398br  M0206cf  M0206cr  M0192f  M0192r | 5' CCT TCA CTG CCA TCC TGT AT 3'  5' GGT AGT TGT TGT GAG CGT AGT C 3'  5' CCC CGA TCC ATT TCA TAG 3'  5' ATC CTG ATG CTG ACT TGA CA 3'  5' TTA TTT GTA CTG GTT TTT TTG TGT 3'  5' GTT CAG GAT AAG CGA GTG AC 3'  5' ATG GTG AAG GTC GGA GTG A 3'  5' AAC ATC CAC TTT GCC AGA GTT A 3' | 86.3  82.2  78.6  83.9 | 90  160  87  84 |


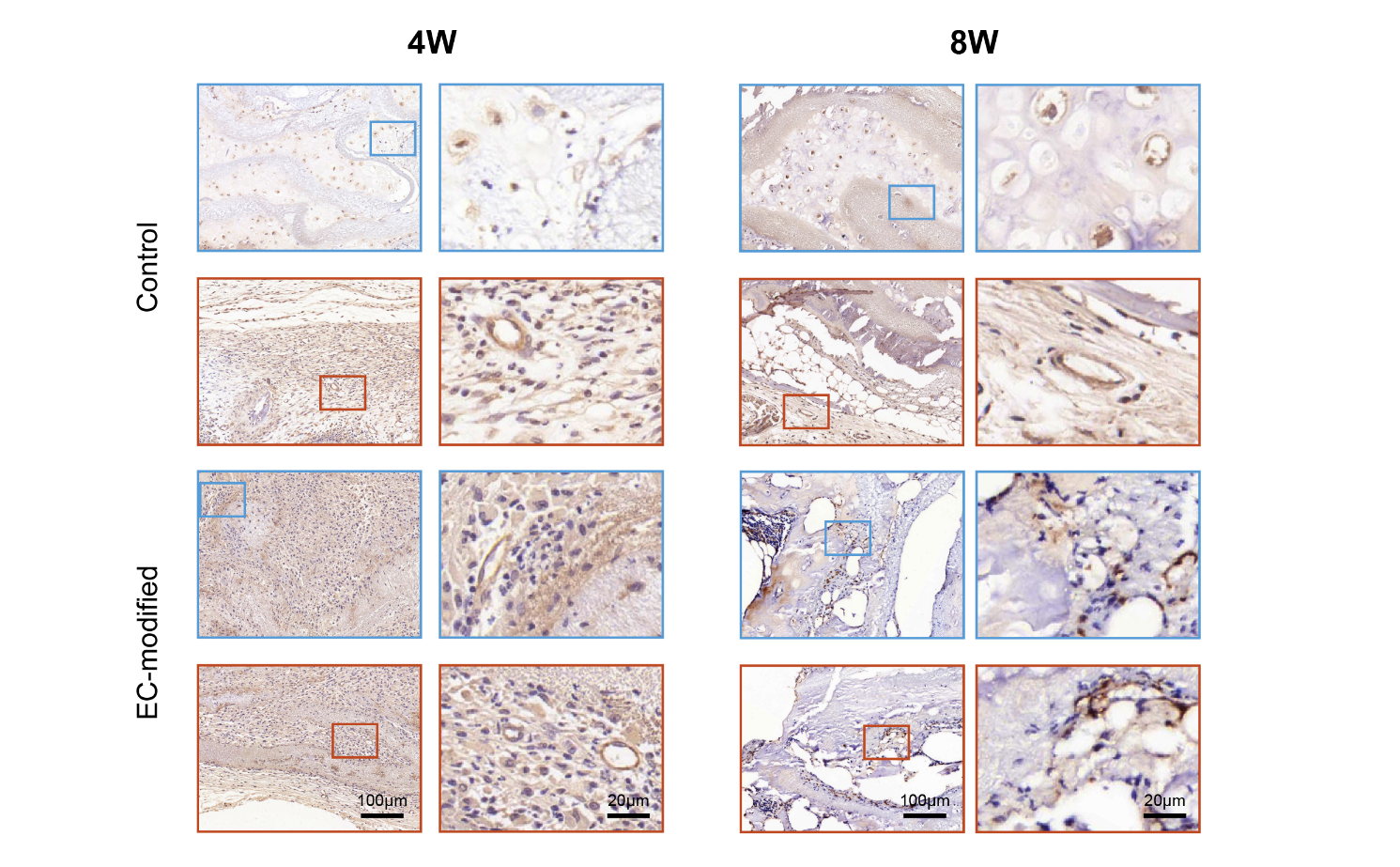


**Figure S1. CD31 immunohistochemical analysis of tissue sections from control and EC-modified groups (4 and 8 Weeks).**

Representative CD31 immunohistochemical staining images showing tissue sections from control and EC-modified groups at 4 weeks (4W) and 8 weeks (8W) post-treatment. Each condition is presented with a low-magnification overview (left panels, scale bar: 100μm) and corresponding high-magnification images of the boxed regions (right panels, scale bar: 20μm).
